# Vitrification freezing of large ovarian tissue in the human body

**DOI:** 10.1186/s13048-019-0553-x

**Published:** 2019-08-22

**Authors:** Qian Zhao, Ying Zhang, Ke Su, Xiao-Wan Wang, Pan-Pan Hai, Bing Han, Ai-Ping Bian, Rui-Xia Guo

**Affiliations:** Department of gynecology, The First Affiliated Hospital of Zhengzhou University, 1 Jianshe Road, Zhengzhou City, Henan 450052 People’s Republic of China

**Keywords:** Ovary, Cryopreservation, Transplantation, Heterologous, In situ nick-end labeling, Ki-67 antigen

## Abstract

**Background:**

This study aims to carry out the vitrification freezing of a large ovarian tissue in the human body, and evaluate its feasibility.

**Results:**

A total of 18 ovarian tissues in the human body were selected, and each tissue was cut into three large ovarian cortex slices. These tissues were randomly divided into three groups: vitrification freezing group (group A), programmed freezing group (group B), and fresh control group (group C). Then, the morphological analysis and apoptosis detection of each ovarian tissue was carried out, followed by the recycling of ovarian tissues at three weeks after the heterotransplantation of nude mice, in order to detect the follicle preservation conditions. The immunohistochemistory method was applied to detect the follicle activity. In comparing the proportion of primordial follicle with normal morphology after unfreezing between group A and group B, the difference was not statistically significant (*P* > 0.05). Furthermore, the incidence of follicle apoptosis in group A and group B was higher than that in the group C (*P* < 0.05). However, when comparing between group A and group B, the difference was not statistically significant (*P* > 0.05). The interstitial cell apoptosis rate in group A was lower than that of the group B, showing that the difference was statistically significant (*P* < 0.05).

**Conclusions:**

Compared with programmed freezing, the vitrification freezing of large ovarian tissues in the human body was feasible to a certain extent. This can be used as an alternative scheme to realize the freeze preservation of ovarian tissues in the human body.

## Background

In recent years, the progress of tumor treatment technology has greatly improved the long-term prognosis of malignant tumor patients. More and more females at childbearing age and juvenile female patients have survived from this. However, there were some problems after tumor radiotherapy and chemotherapy, such as ovarian dysfunction and dysgenesis, which has become a top concern for them [1]. The vitrification cryopreservation of ovarian tissues is a kind of new technology secondary to embryo freezing and oocyte cryopreservation, with the potential for preserving female reproductive function. Through the quick cooling rate, it can be used to induce internal and external cells to the vitrification state, avoiding the formation of ice crystals. Therefore, it is a kind of technology that is easy to perform for the simple and quick preservation of complex constructions [2]. At present, the application of the vitrification freezing method to realize the freeze preservation of ovarian tissues in the human body has been successively reported [3, 4]. However, compared with the vitrification freezing of the embryo and oocyte, there is still no standard scheme for vitrification freezing of ovarian tissues. At present, the vitrification freezing of ovarian tissues mainly adopts the small cortex slice (thickness: approximately 1 mm, area: ≤1 cm^2^) for freeze preservation, and there are few reports on the vitrification freezing of large ovarian tissues in the human body. The present study discussed the feasibility of vitrification freezing for large ovarian tissues in the human body (area: approximately 180 mm^2^) by comparing this with the traditional slow programmed freezing method, and establishing a nude mice heterotransplantation model, providing a new scheme for fertility preservation in tumor patients.

## Materials and methods

### Specimen source and processing

**Fig. 1:**
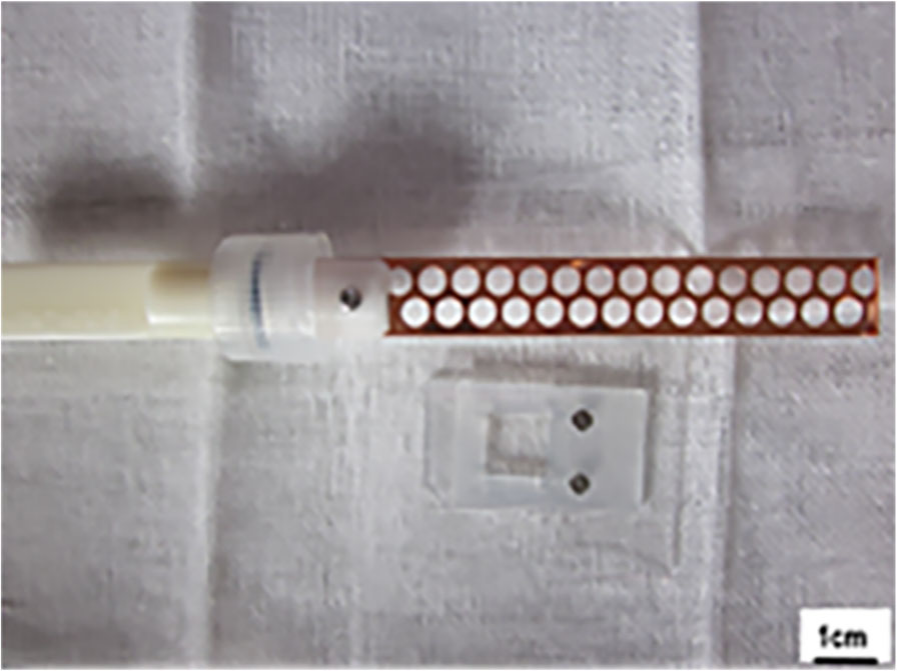
Vitrification freezing carrier and template

**Fig. 2:**
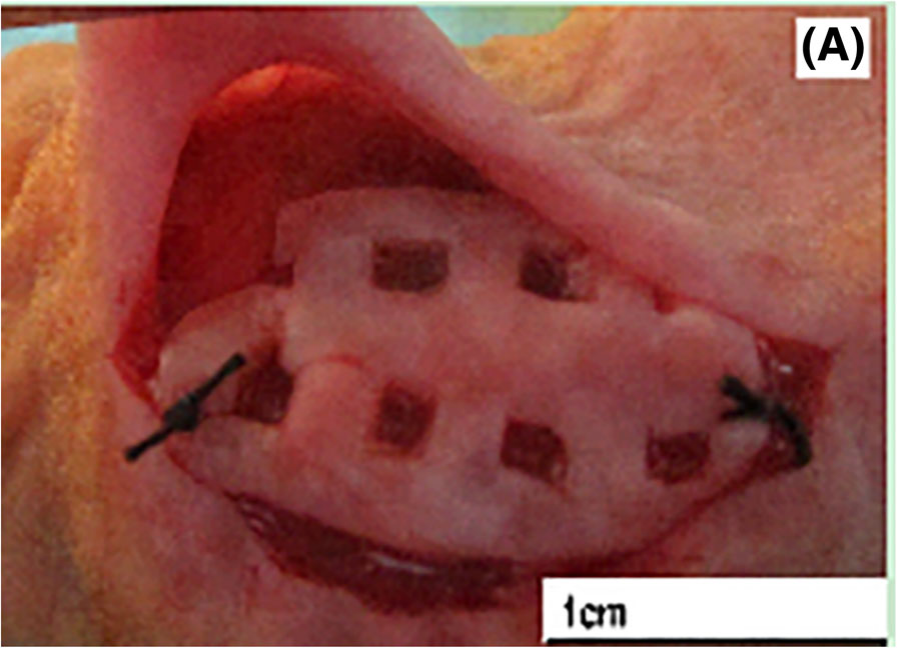
General observation of the ovarian tissue slice before transplantation

A total of 18 patients, who underwent laparoscopic or open operation to perform the ovariectomy at the Department of Gynecology, the First Affiliated Hospital of Zhengzhou University from December 2013 to September 2015, were included in the study. Among these patients, and through pathological diagnosis, twelve patients had cervical cancer, three patients had endometrial cancer, and three patients had breast cancer. After the quick freezing biopsy of ovarian tissues was conducted during the operation, it was proven that no ovarian metastasis occurred, and part of ovarian tissue cortex was taken as the specimen of the present study. The average age of these patients ranged within 37.9 ± 6.4 years old, and these patients had no endocrine disease and hormone drug administration history, or chemotherapy and radiotherapy history. The present study obtained the approval and consent of the Medical Ethics Committee of Zhengzhou University, and all patients provided a signed informed consent. After the collected ovarian tissues from the human body were placed in Dulbecco’s phosphate buffered saline (DPBS) preheated below 4 °C, these were quickly transferred to the laboratory. Then, the tissues were cleaned and the bloody water was removed. Afterwards, the template used for cutting (Kitazato BioPharma, Fig. 1) was put in the ovarian surface, and the blade was used to accurately split the ovarian cortex into three tissue slices at a size of approximately 1 mm (thick) × 18 mm (long) × 10 mm (wide) and one tissue slice at a size of 1 mm (thick) × 10 mm (long) × 10 mm (wide). Four ovarian tissues were respectively taken from every patients, and the specially designed cutting tool was used to cut the ovarian cortex, in order to make the ovarian tissue slice surface evenly distribute to the square holes, which had a size of 2 × 2 mm. The spacing between holes was 2 mm (Fig. 2). After being cut, these slices were randomly divided into the following groups: vitrification freezing of large ovarian tissue group (group A), programmed freezing group (group B), and fresh control group (group C), and vitrification freezing of small ovarian tissue group (group D).

### Main reagents and animals

The main reagents include human serum and fetal calf serum (COOK), dimethyl sulfoxide (DMSO), ethylene glycol (EG), saccharose and penicillin-streptomycin, and other reagents (Sigma), as well as the Ki-67 antigen (Abcam), SP kit (Bioss), and TUNEL detection kit (Roche). There were a total of 54 animals, which comprised of 5–6 year-old female nude mice with a body mass of 18–22 g (Beijing Vital River Laboratory Animal Technology Co., Ltd.). The experimental animal quality conformity certificate was SCXK (J) 2014–0010. Upon approval of the Animal Ethics Committee, these mice were randomly divided into three groups: group A, group B, and group C. Each group comprised of 18 mice.

### Vitrification freezing and programmed freezing process

The vitrification freezing method applied by Kagawa et al. [5] was adopted for improvement, and the processed tissue slice was balanced for five minutes with basic fluid, which contained DPBS with 20% fetal calf serum, under room temperature. Then, this was transferred to the balanced fluid, which contained basic fluid with 7.5% ethylene glycol and 7.5% dimethyl sulfoxide, in order to balance for 20 min. Afterwards, the tissue slice was transferred to the refrigerating fluid, which contained basic fluid with 15% ethylene glycol and 15% dimethyl sulfoxide, in order to balance for 15 min. After the ovarian tissue slice was taken out from the refrigerating fluid, the excess refrigerating fluid was removed, and the tissue slice was placed in a metal carrier preheated in liquid nitrogen (cryotissue, Kitazato BioPharma; Fig. 1). Then, the tissue slice was placed in the cryovial, and preserved in a liquid nitrogen container. After one week, the cryovial was taken out, the metal carrier was taken out from the cryovial in a quick manner, and this was quickly placed in a culture dish containing 1 MS of fluid (basic fluid + 1 mol/L of saccharose) preheated under 37 °C, 0.5 MS of fluid (basic fluid + 0.5 mol/L of saccharose), 0.25 MS of fluid (basic fluid + 0.25 mol/L of saccharose), and basic fluid, in proper order, in order to gradually dilute and remove the cryoprotective agent. The balance time was five minutes. The programmed freezing and unfreezing process was based on the program freezing method recommended by Gosden [6].

### Heterotransplantation

The tissues were cut at a size of approximately 2 × 2 mm from the unfrozen tissue slice in group A 、group B and group D, and the fresh tissue slice in group C, respectively. After fixation in 10% neutral formalin solution, hematoxylin and eosin (H&E) staining and apoptosis detection were performed. For residual tissues, a heterotransplantation operation was conducted. After successful anesthesia, the back skin of nude mice was routinely disinfected, and a blunt dissection was made on the subcutaneous tissue at the back and in the fascia. Afterwards, ophthalmic forceps were used to clamp the unfrozen/fresh human ovarian tissue, and transplant this in the subcutaneous trauma clearance. A 4–0 silk thread was used to fix the mark before the skin was sutured. After the operation, 40,000 U/d of penicillin was injected into the abdominal cavity to prevent infection for a total of three days. At five months after transplantation, the neck-off method was used to kill the nude mice, and the transplanted ovarian slice was taken out to observe the size, morphology and blood supply, as well as detected the follicle activity, of the recycled ovarian tissue slice.

### Morphological analysis and apoptosis detection of ovarian tissues after cryopreservation

The fresh or frozen recovered ovarian tissues were routinely dehydrated, and the xylene transparent and paraffin embedding method was used to carry out the continuous slicing at 4 μm, followed by the H&E staining or apoptosis detection at an interval of 10 slices. After the H&E staining, the Gougeon standard [7] was referred to observe the follicle morphology, and count the proportion of the morphology, and normal and abnormal primordial and primary follicles in each slice. TdT-mediated dUTP nick-end labeling (TUNEL) was adopted to detect the follicle and interstitial cell apoptosis conditions. The operation followed the steps in the instructions of the TUNEL detection kit (Roche). When the cell nucleus was deeply stained with brownish yellow through observation, positive staining was recorded. When the oocyte in the primordial follicle was positively stained and/or the positive staining of the granule cell exceeded 50%, the apoptosis positive primordial follicle [8] was recorded, and Image-Pro Plus 6.0 was adopted to carry out the semi-quantitative determination and judgment of the interstitial cell apoptosis area. That is, the interstitial cell apoptosis positive rate = staining positive interstitial cell area/total tissue area × 100%. The experiment was repeated for three times. The researcher counting the primordial follicles and apoptosis of the primordial follicle and interstitial cells were blind to the type of the group.

### Determination of follicle activity after transplantation

The recycled transplant of these three groups was routinely dehydrated. The xylene was transparent, and the paraffin embedding method was used to carry out the continuous slicing at 4 μm. Then, the immunohistochemistory method was used for staining at an interval of 10 slices. The anti-Ki-67 antibody (Abcam) was used to dilute for 150 times, and the operation followed the steps in the instructions for SP detection kit (Bioss). Each slice randomly selected 10 fields of vision. Under a 400× microscope, the count was observed. The staining intensity scoring standard was as follows: no staining, 0 point; yellow, 1 point; brownish yellow, 2 points; yellowish brown, 3 points. The scoring for the staining distribution in positive cells was as follows (0–100%): no distribution, 0 point (cell staining was negative); dispersed distribution, 1 point (< 10%); concentrated distribution, 2 points (10–50%); diffused distribution, 3 points (> 50%). The sum of the above two items of scores were as follows: 0–3 points (−), 4 points or above (+). These were used to calculate the positive rate. The experiment was repeated for three times.

### Statistical method

SPSS 19.0 statistical software was used for the analysis. Quantitative data were expressed as mean ± standard deviation. Comparisons among the three groups were performed using one-way analysis of variance, while comparisons between two groups were performed using the Bonferroni method (inspection level α´ = 0.0167). Qualitative data were expressed in percentage. Comparisons among the three groups were performed using the *X*^*2*^-test, while the rate comparisons among these three groups were performed using the Kruskal-Wallis rank-sum test. *P* < 0.05 was considered statistically significant.

## Result

### Morphological analysis of the ovarian tissue

**Fig. 3:**
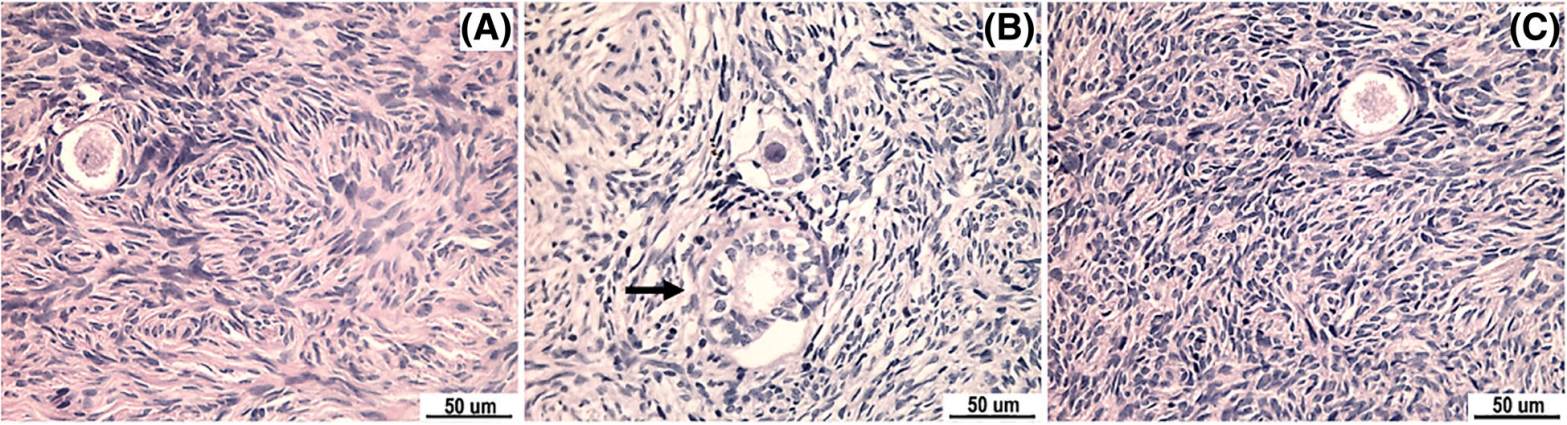
Comparison on follicle morphology among the three groups (H&E, × 400). (**a**) The morphology of a normal primordial follicle in the vitrification freezing group manifested that the follicle morphology was round, the distribution of granule cells was relatively even, and the oocyte envelope was complete. (**b**) The morphology of an abnormal primordial follicle and secondary follicle (arrow) in the programmed freezing group manifested that the distribution of granule cells was not even, the morphology was irregular, and the oocyte envelope was incomplete. (**c**) Morphology of a normal primordial follicle in the fresh group

By comparing the frozen human ovarian tissue with the fresh control group under an optical microscope, the morphology of most primordial follicles was basically in a well-preserved state (Fig. 3). In comparing the proportion of primordial follicles with normal morphology in group A, B and C, the difference was statistically significant (*X*^*2*^ = 6.434, *P* < 0.05). In further comparing between two groups, the proportion of primordial follicles with normal morphology in group B was lower than that in the group C (*X*^*2*^ = 6.417, *P* < 0.0167). In comparing among group A and B and C, the difference was not statistically significant (the *X*^*2*^ values were 0.804 and 2.729, respectively; *P* > 0.0167). In comparing the proportion of primary follicles with normal morphology among these three groups, the difference was statistically significant (*X*^*2*^ = 12.909, *P* < 0.01). In addition, the proportion of primary follicles with normal morphology after freezing in the vitrification freezing group significantly decreased, but was higher than that in the programmed freezing group (*X*^*2*^ = 13.022, *P* < 0.0167) (Table 1).

**Table 1:** The proportional change of normal morphology and abnormal morphology in each group Number of case(%)

Group	*n*	Primordial follicles			Primary follicles		
		Total	Normal	Abnormal	Total	Normal	Abnormal
Vitrification freezing group (A)	18	113	90 (79.6)	23 (20.4)	33	17 (51.5)	16 (48.5)
Programmed freezing group (B)	18	102	76 (74.5)	26 (25.5)	36	12 (33.3)	24 (66.7)
Fresh control group (C)	18	128	112 (87.5)	16 (12.5)	31	24 (77.4)	7 (22.6)
*χ*^2^ (*P*)			6.434 (0.040)			12.909 (0.002)	
*P′*			0.0167			0.0167	

### Comparison on the incidence of apoptosis of the primordial follicle and interstitial cells among the three groups

**Fig. 4:**
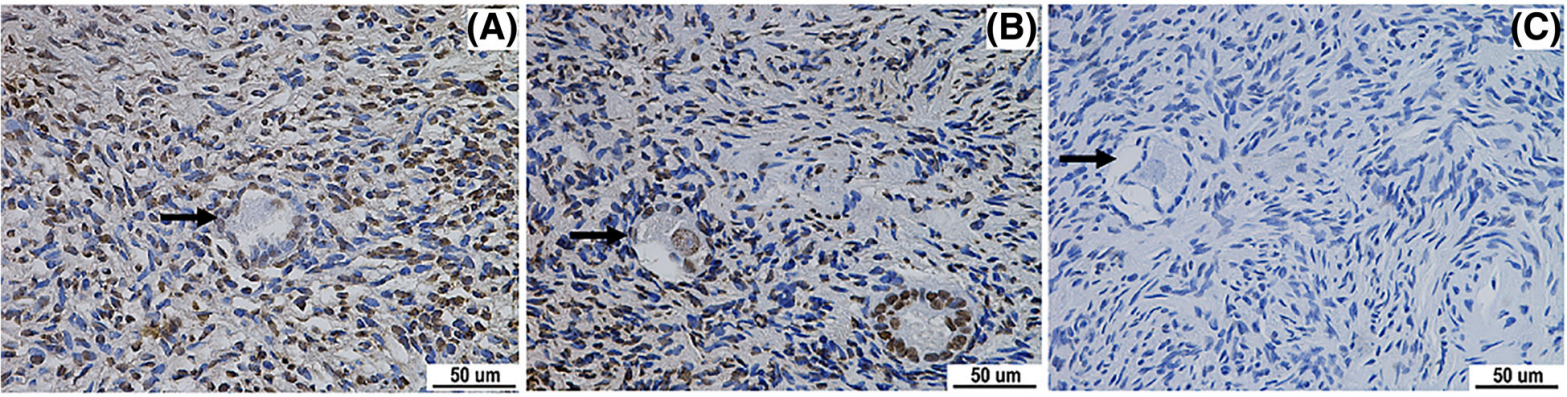
Comparison on apoptosis staining between the freezing groups and fresh groups (TUNEL, × 400). (**a**) Apoptosis positive primordial follicle in the vitrification freezing group. (**b**) Apoptosis positive primordial follicle and primary follicle in the programmed freezing group. (**c**) Apoptosis negative primordial follicle in the fresh control group

By analyzing the apoptosis of the primordial follicle and interstitial cells in the ovarian cortex of these three groups under an optical microscope, it was found that apoptosis positive primordial follicles in the fresh control group were rare, and that interstitial cells were dispersed by staining (Fig. 4). By comparing the incidence of apoptosis of the primordial follicle in these three groups, the difference was statistically significant (*X*^*2*^ = 16.508, *P* = 0.000). When this was compared to the fresh control group, the incidence of apoptosis of the primordial follicle in the vitrification freezing group and programmed freezing group significantly increased, showing that the difference was statistically significant (*X*^*2*^ values were 11.681 and 15.238, respectively; *P* = 0.000). However, for the comparison between group A and group B, the difference was not statistically significant (*X*^*2*^ = 0.278, *P* = 0.592; Table 2). However, the analysis on the apoptosis of ovarian interstitial cells revealed that the apoptosis rate of interstitial cells was significantly lower in the vitrification freezing group (30.16%) than in the programmed freezing group (39.83%), and these were higher than that in the fresh control group (6.47%). The difference was statistically significant (*P* < 0.05).

**Table 2:** Comparison of the incidence of apoptosis of the primordial follicle Number of case (%)

Group	*n*	Number of follicles	Positive	Feminine
Vitrification freezing group (A)	18	138	36 (26.1)	102 (73.9)
Programmed freezing group (B)	18	131	38 (29.0)	93 (71.0)
Fresh control group (C)	18	144	15 (10.4)	129 (89.6)

### Transplant recycling and morphological analysis

**Fig. 5:**
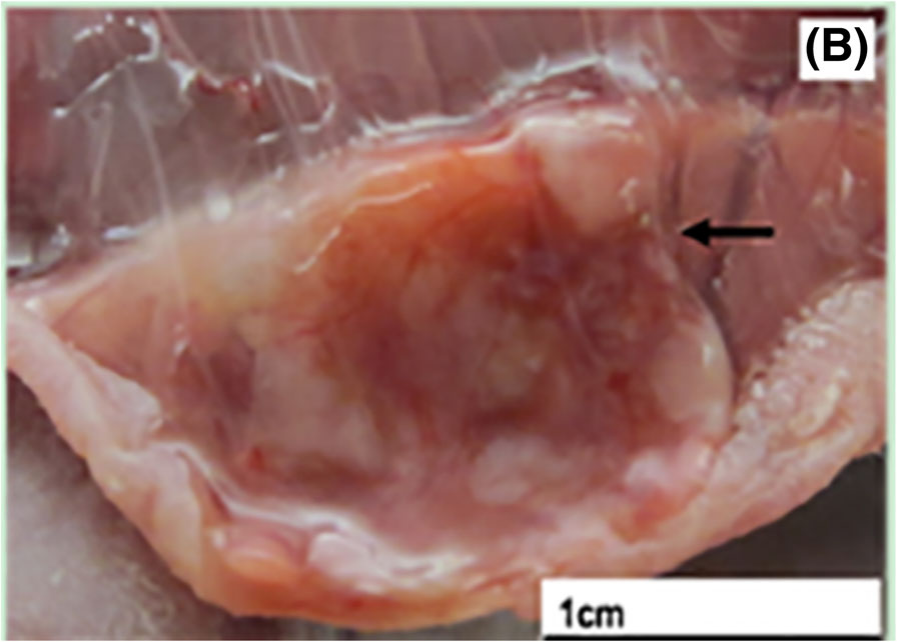
General observation of the ovarian tissue slice after transplantation

The transplant recycling rate was 100% and the volume of transplants that partially survived relatively shrunk, when compared to before. In addition, for most recycled transplants, the appearance was bright and ruddy, and the surface coating contained thin-layer fibrous tissues. Meanwhile, many blood vessels were attached in the junction of transplants (Fig. 5). The manifestations of recycled ovarian tissues under an optical microscope revealed that the partial follicles shrunk to form the vacuolation, the follicle density significantly declined, and interstitial cells decreased with tissue fibrosis. Meanwhile, many newly born capillaries formed in the junction of the transplant and the host. The densities of primordial follicles in group A, B and C were 0.83 ± 1.10/mm^2^, 0.72 ± 0.90/mm^2^ and 1.06 ± 1.06/mm^2^, respectively, and the difference was not statistically significant (*P* > 0.05).

### Analysis of follicle activity in the transplant

The follicle and interstitial cell in the recycled ovarian tissue were dispersed in the expressed Ki-67 antigen, the expression of primordial follicles was rare, and the positive expression was mainly embodied in oocytes and granule cells in the follicle during the growing period. In comparing the positive rate of follicles in group A (15.3% [13/85]), group B (14.6% [12/82]), and group C (11.6% [8/69]), the difference was not statistically significant (*X*^*2*^ = 0.476, *P* > 0.05).

## Discussion

### Clinical value

The preservation effect for vitrification freezing of ovarian tissues was influenced by ovarian tissue size, refrigerant concentration, refrigerant balance time, the carrier system applied and other multiple factors. These induced the refrigerating fluid evenly penetrate into the ovarian tissue and ensure the consistency of the freezing rate of each tissue. Furthermore, the cryopreservation of ovarian tissues mainly adopts cryopreserved small tissues (area: < 1 cm^2^, thickness: 1 mm) [9]. However, the tissue slice volume was not too small, in order to avoid the missing or damaging of excessive follicles during cutting, which would thereby produce many unavailable tissue slices. Some studies have reported that the freezing and thawing process of ovarian tissues can cause a loss of 7% of follicles, and after transplantation and before blood supply reconstruction, a loss of more than 60% of follicles may be caused, reducing the practical significance for cryopreservation and transplantation of small ovarian tissues [10]. Poirot et al. [11] considered that the freezing of large ovarian tissues can acquire more follicles, protecting the preantral follicle with its large volume, and guaranteeing the structure in the tissue to better support follicle growth. Gook et al. [12] considered that after cutting the ovarian tissue into slices with the size of 2.0–5.0 mm, the influence on freezing was not significant, and preantral follicles with a large volume were also protected. Anerdsen et al. [13] transplanted a large ovarian tissue (close to 1/3 of the whole ovarian tissue) to one patient with the acute lymphoblastic leukemia, and made the ovarian tissue function continuously for seven years, and even longer. A certain amount of follicles is the basis to maintain ovarian function, and for patients with ovarian function failure, who underwent high-dose radiotherapy and chemotherapy. Theoretically speaking, it is very beneficial to transplant more ovarian cortexes with follicle growth activity [13]. Hence, in order maintain ovarian function after transplantation for a long time, the cortex size should be increased as far as possible under the premise of considering the freezing effect and follicle survival rate.

### Feasibility analysis

The key for vitrification freezing is to make the refrigerant penetrate into the ovarian tissue, and effective tissue penetration is the premise for tissues to smoothly enter into the vitrification state during cooling. In the present experiment, the tissue slice was trimmed to form an even hole clearance, and the physical diffusion principle was used to realize the effective penetration of the refrigerant and reduce balance time. In this vitrification freezing scheme, the cryotissue was used as a carrier to bear the large ovarian cortex slice. Meanwhile, in this scheme, the tissue was directly placed in a metal carrier that has been precooled in liquid nitrogen, in order to avoid heat conduction block due to the formation of bubbles generated from the evaporation of liquid nitrogen, and minimize the cryoprotective agent required for frozen tissue, thereby improving cooling speed and promoting the formation of a vitrification state [14]. This experiment cryopreserved a large area of ovarian tissue slice, but the ice crystal was not obviously formed during vitrification freezing and after unfreezing. Approximately 79.6% of primordial follicles in the vitrification freezing group maintained a normal morphology, which was slightly lower than the normal morphology rate in fresh ovarian tissues (87.5%). However, such rate corresponded to the normal morphology rate of 70–90% of primordial follicles after freezing, as reported in other literatures [15–17]. This proves that after the tissue slice was trimmed, the refrigerant was evenly penetrated to prevent the ovarian tissue from forming an obvious ice crystal during the transformation to the vitrification state, thereby leading to a good follicle preservation effect. At the same time, the main preservation object of ovarian tissue freezing includes the primary follicle, accounting for 20–30% of the total number of follicles, in addition to the primordial follicle. Through the morphological analysis of the follicle, by comparing with programmed freezing, vitrification freezing has a significant advantage on the cryopreservation of the primary follicle.

Due to the limitations of the morphological analysis in the evaluation of ovarian tissue development potentiality, the present experiment adopted the analysis of the apoptosis index to compare the effects of different freezing methods on the preservation of ovarian development potentiality. It was found that the follicle apoptosis rate was similar among freezing groups, while for the apoptosis rate of the primordial follicle, the difference was not significant. Meanwhile, the incidence of apoptosis of the interstitial cell in the vitrification freezing group was lower than that in the programmed freezing group, prompting that the vitrification freezing method can be used to better protect ovarian interstitial cells, providing better tissue samples for the clinical application of ovarian tissues after freezing and thawing. The apoptosis was closely correlated to the normal development of human ovarian tissues and its function. It is undeniable that vitrification freezing can cause a certain degree of damage on follicles, which may be due to the high concentration of the cryoprotective agent.

The present experiment selected subcutaneous tissues in the back of nude mice as the transplantation part. This was due to its loose nature and relatively rich blood supply, which is beneficial for the survival of the transplants. Denschlag et al. [18] reported that a large ovarian tissue slice (diameter: approximately 2 cm, thickness: approximately 2–3 mm) can survive after autoplastic heterotopic transplantation without vascular anastomosis. However, the blood supply of ovarian tissues after transplantation completely relies on the growth of peripheral capillaries without the help of vascular anastomosis, and excessive long or thick tissues can make the blood vessels of an animal subject spend more time entering into the tissue by means of peripheral growth. The longer the new vessels completely cover the tissue, the larger the ischemic injury becomes, and will cause ischemic injury to the tissue at the early stage of transplantation, and influence the development of follicles. The present experiment revealed that the volume of partial transplants shrunk, and most follicles had locking, tissue fibrosis, obviously declined density, and other conditions. These were consistent with the phenomenon on the loss of many follicles due to ischemia reperfusion injury in the ovarian transplantation test on mice performed by Liu et al. [19]. However, in the present experiment, there were many new vessels in the junction of the transplant and the host, and under an optical microscope, rich capillaries formed. Meanwhile, the follicle distribution was relatively rich, showing that after the ovarian tissue slice was trimmed through holes, the new vessels formed by the host could enter the tissue through these evenly distributed holes, reducing the ischemic injury of the tissue, and providing a possibility for survival and further development of follicles.

The proliferating cell nuclear antigen Ki-67 antigen expression was located in granule cells, and the oocyte cytoplasm and nuclei, and it is an index to evaluate the cell proliferation state. Its expression is positively correlated to the cell proliferation in the cell cycle of G1, S, G2 and M, other than G phase. Furthermore, it can be used as a specific marker of cell proliferation to evaluate follicle activity, and the proliferation of the granule cells is usually considered as an early marker of primordial follicle activation [20]. The present experiment revealed that the expression of the Ki-67 antigen in the transplant was mainly embodied in the surviving follicle during the growth period, while the expression of primordial follicles was rare, which was mainly due to the primordial follicle in the dormancy period. In comparing the follicle positive rate of the Ki-67 antigen expression in the transplant among these three groups, the difference was not statistically significant. This might be associated to statistical error due to loss of follicles after the transplantation. However, in the vitrification freezing group, the positive staining of follicles during the growing period was identified, indicating that ovarian tissues after vitrification freezing continued to have a potential for proliferation in vitro. Furthermore, whether there is tissue and cell activity after human ovarian tissues are frozen and thawed, the activated cell mass, follicle growth, and its development ability are constrained. Other issues need to be further researched to carry out further confirmations and interpretations.

## Conclusion

In summary, the present experiment adopted the vitrification freezing method to preserve large ovarian tissues, and compared this with the classic programmed freezing method, in order to obtain a better cryopreservation effect, when compared to the programmed freezing scheme, proving the possibility of vitrification freezing for large ovarian tissues. However, since the follicle density and activity determination after the transplantation failed to manifest an obvious advantage, this indirectly reflects that this scheme needs to be further researched and perfected. Thus, this is expected to be used for the cryopreservation of ovarian tissues, providing a scheme for the ultralow temperature cryopreservation of ovarian tissues in female tumor patients.

## Data Availability

Not applicable.

## Abbreviations

DMSO: Dimethyl sulfoxide
EG: Ethylene glycol
H&E: Hematoxylin and eosin
TUNEL: TdT-mediated dUTP nick-end labeling
